# Pericardial Effusion After Pediatric Cardiac Surgeries: A Single Center Observation

**DOI:** 10.5812/cardiovascmed.4601

**Published:** 2012-11-01

**Authors:** Mohammad Dalili, Hassan Zamani, Mohammadyousef Aarabi-Moghaddam

**Affiliations:** 1 Department of Pediatric Cardiology, Rajaie Cardiovascular Medical and Research Center, Tehran University of Medical Sciences, Tehran, IR Iran; 2 Babol University of Medical Sciences, Babol, IR Iran

**Keywords:** Postpericardiotomy Syndrome, Pericardial Effusion, Echocardiography

## Abstract

**Background:**

Development of fibrinous pericarditis after pericardiotomy is a well-recognized reaction. Within a few post-operative days, the inflammated surface of pericardium begins to fuse to the overlying sternum.

**Objectives:**

Our aim was to assess the prevalence, risk factors, time course and therapy response of pericardial effusion (PE) after cardiac surgeries in children.

**Patients and Methods:**

PE occurrence was assessed prospectively in 486 children who underwent cardiac surgery for congenital heart diseases by serial echocardiography. Clinical manifestations were observed and response to different therapies was analyzed.

**Results:**

The prevalence of PE was about 10% for all cardiac surgeries. Symptoms were exclusively seen in patients who had moderate to large effusions. The mean onset of pericardial effusion was 11 (± 8) days after surgery procedure, with 87 % (42 of 48) of cases being diagnosed on or before day 13 after operation. The prevalence of effusion after Fontan-type procedures and AVSD repair (29 %, 5 of 17 for both) was significantly higher than other types of cardiac surgeries. Aspirin administration was effective in 77 % and prednisone in 90 % of the cases.

**Conclusions:**

PE may be developed as late as weeks after cardiac surgeries. PE after palliative cardiac surgeries is not uncommon. Low doses of aspirin and corticosteroids are usually effective for treating this complication.

## 1. Background

Development of fibrinous pericarditis after pericardiotomy is a well-recognized reaction. Within a few post-operative days, the inflamed surface of pericardium begins to adhere fuse to the overlying sternum. When this occurs, exuding of blood from the pericardial surface can result in the insidious development of postoperative pericardial effusion (1). Pericardial effusion occurs most commonly after corrective cardiac surgeries but may be observed in all types of surgeries involving the pericardial sac, by direct or indirect irritation (2, 3).Nonetheless, data on its prevalence and time course after surgeries for congenital heart diseases are limited. Furthermore, risk factors which predispose its development toward fusion after undergoing cardiac surgery in children and teenagers with congenital heart diseases remained unknown. 

## 2. Objectives

In this study, we tried to determine the prevalence and time course of pericardial effusion after different types of heart surgeries in congenital heart diseases.

## 3. Patients and Methods

A total of 519 consecutive subjects including infants, children and teenagers, who have undergone a cardiac surgery for congenital heart diseases were recruited during one year. Serial two-dimensional echocardiography was performed on first, third, fifth and tenth day postoperatively. In those patients who developed PE or patients who stayed in hospital longer, echocardiography was repeated each three days until discharge. Follow-up echocardiography was performed on all patients after one month. Echocardiography was performed using a Vivid three ultrasound machine (GE, USA) by trained pediatric cardiology fellows. Pericardial effusion was assessed in the standard parasternal short axis, long axis, apical four chamber, and subcostal echocardiography views. The amount of pericardial effusion (PE) was graded according to the dimension of maximum separation between pericardium and epicardium at diastole; dimensions less than five millimeters (mm) were considered as mild, six to 15 mm as moderate, and more than 16 mm as severe. The distribution pattern of effusion was described as circumferential or loculated. The following data were collected: demography, type of cardiac defect, type of surgery performed, postoperative use of anticoagulants and diuretics, and occurrence of pericardial effusion. In patients who developed pericardial effusion, the timing of onset and duration of effusion, their clinical presentation, and the treatment received were noted. The demographic, clinical, and perioperative variables between patients who developed pericardial effusion and those who did not were compared using unpaired Student’s t test, Mann Whitney U, Fisher’s exact, and Chi-square test. Logistic regression model was used to identify risk factors predisposing to develop pericardial effusion. Variables as described above were entered into the multivariate model. A probability value of P < 0.05 was considered significant. All statistical analyses were performed using SPSS 15 for windows (SPSS Inc. : Chicago, Illinois).

## 4. Results

Five hundred and nineteen patients (237 males) were recruited. Three hundred thirty four surgeries have been done with mid-sternotomy and the other 185 were done via thoracotomy. Thirty three patients did not survive surgery because of different causes, but none of them expired due to pericardial effusion; the remaining 486 patients were included. In all cases, pericardial sac had been opened partially or totally during surgery. Pleural cavity had been opened only for those surgeries which were done via sternotomy. All patients had at least one chest drain post-operatively. In operations that were done with thoracotomy (Modified BT shunt, Glenn shunt, COA repair, PDA closure, PA banding, Atrial Septectomy, ASD repair and their combinations) only one chest drain has been used. For the other surgeries done via mid-sternotomy two chest drains were used; one for the mediastinum and the other for the pleura that was opened. Rare cases had three drains. In one case re-exploration of mediastinum was done because of excessive bleeding. Mean age of the patients was 3.7 years (range three months to 18 years) and the mean weight was 17 kg (range 3.2 – 66 kg). Thirty three of patients died in operation room or in the first day post-operation. The surgeries performed and the number (percent) of patients who developed PE after each type of surgery is shown in Table 1. Of 486 survived patients, 48 (9.9%) patients developed pericardial effusion postoperatively. The amount of effusion was mild in 22, moderate in 17, and severe in nine patients. The effusion was circumferential in 45 of 48 (94%) at first presentation. Of the three loculated effusions, two were small and one was moderate in size.

**Table 1.** Types of Surgeries and Relative Occurrence Rate of PE

Type of Surgery	Number of patients	Number of patients with PE ^a^ , (%)
Modified BT ^a^ (Gore-Tex) Shunt	80	1 (1)
Glenn shunt	8	4 (50)
COA ^a^ repair +/- PDA closure	18	0 (0)
PA ^a^ banding +/- PDA closure	56	7 (12)
PDA ^a^ L&D	15	0 (0)
Atrial Septectomy +/- PA banding	27	1 (4)
ASD ^a^ repair	33	1 (3)
VSD ^a^ repair	74	9 (12)
TAPVC ^a^ repair	8	0 (0)
Rastelli type operation	2	0 (0)
Vulvar repair	24	2 (8)
TFTC ^a^	69	10 (14)
TF + AVSD ^a^ repair	2	0 (0)
Modified Fontan operation	17	5 (29)
AVSD repair	17	5 (29)
AP ^a^ window repair	4	0 (0)
ASD + VSD repair	8	2 (25)
Senning operation	10	1 (10)
Arterial switch	4	0 (0)
Truncus arteriosus early repair	2	0 (0)
Valve replacement	8	0 (0)
Total	486	48 (10)

^a^ Abbreviations: AVSD: Trio-Ventricular Septal Defect; ASD: Atrial Septal Defect; AP: Aorto-Pulmonary; BT: Blalock-Taussig ; COA: Coarctation of Aorta; VSD: Ventricular Septal Defect; PA: Pulmonary Artery; PDA: Patent Ductus Arteriosus; PE: Pericardial Effusion; TAPVC: Total Anomalous Pulmonary Venous Connection; TFTC: Total Correction of Tetralogy of Fallot

There was no significant difference between genders in occurrence of PE. The prevalence of pericardial effusion after Fontan-type procedures and AVSD repair (29 %, 5 of 17 for both) was significantly higher than other types of cardiac surgeries (P = 0.03). Furthermore, pericardial effusions that developed after Fontan-type procedure were at least moderate to severe. Postoperatively, a greater pericardial drain output within the first four hours tended to be associated with a higher risk of developing later effusion (P = 0.045). Warfarin given to 33 patients including those who had undergone valve replacement (n = 8), Fontan-type procedures (n = 17), and Glenn shunt (n = 8), also conferred a significant risk factor of developing pericardial effusion (27.2 % vs. 9.9 %). The onset of pericardial effusion accumulation was on day 11 (± 8) after surgery and PE diagnosis was made on before day 13 in 87 % (42 of 48) of the patients.

Clinical symptoms or signs were present in only nine (19 %) patients. These include; malaise in eight patients, dyspnea in eight and chest pain in one patient. Tachycardia was found in six cases and two patients had fever. Only one patient developed classical findings of tamponade including hypotension and pulsus paradoxus. Patients with severe effusion were more likely to be symptomatic than others (23 % vs. 11 %, P = 0.03). All of three loculated effusions were asymptomatic. Radiographic evidence of cardiomegaly was found in 44 (92 %) patients. 

Fluid restriction and a low-dose diuretic ordered for all cases with PE. Three patients initially developed tamponade and underwent surgical drainage. Effusion was resolved in four patients with mild PE without treatment. Anti-inflammatory medications were started in 41 patients, aspirin in 26 and steroids in 15. The physician’s advice did not follow a rule and was not related to the amount of pericardial effusion. ASA was administered 50 - 70 mg/ kg/ day in four divided doses and prednisolone 1mg/ kg/ day in four divided doses orally. Type of treatment and patient response is summarized in Table 2. 38 of 41 (93 %) patients responded to anti-inflammatory medications, while three eventually required pericardial drainage. Mean period of time for effusion to resolve was 13 ± 8 days with aspirin and 7 ± 3 days for corticosteroids.

**Table 2.** Treatment Type and Patient Responses

	Type of Treatment	
	**Aspirin (n = 26)**	**Corticosteroids** ^**a**^ ** (n = 20)**
**Responders**	20 (77%)	18 (90%)
Pericardial Effusion		
Small	13 (50%)	8 (40%)
Moderate	7 (27%)	9 (45%)
Large	0	1 (5%)
**Non-Responders**	6 (23%)	2 (10%)
Additional Treatments		
Surgical Drainage	1 (4%)	2 (10%)
Others (Corticosteroids)	5 (19%)	-

^a^ 15 (75%) as first drug, 5 (25%) ASA non-responders

## 5. Discussion

The prevalence of pericardial effusion after corrective cardiac surgeries has been reported 53 – 85 % in earlier studies (4-6). However, recent studies in both adults and children suggest a decrease in prevalence. Yip et al. (7) reported a 16 % prevalence in 339 adults undergoing surgical repair of atrial septal defect, while, Prabhu et al. (8) reported 13.6 % prevalence in 212 children and teenagers undergoing surgery for congenital heart diseases. Similarly, our study in a large cohort of patients, shows a relatively lower prevalence of pericardial effusion, which may be attributed to improved surgical techniques, myocardial protection, and improved postoperative hemodynamics.

Additionally, our study describes the estimated time for developing pericardial effusion. While pericardial effusion can be detected within the first 13 days of surgery in the majority (87 %) of patients, 13 % of cases would have been missed if routine echocardiographic screening had been confined to this period of time. Symptoms were present in only nine of our patients who developed pericardial effusion and these symptoms were mostly non-specific. This is in contrast with the findings of Clapp et al. (5), who reported that 16 of 21 children with postoperative effusion developed clinical evidence of postpericardiotomy syndrome. The apparent discrepancy may due to serial echocardiographic assessments which allows for early diagnosis, and also administration of anti-inflammatory drugs before appearance of symptoms which may reduce the risk of developing PE. On the other hand, symptomatic patients were more likely to have a more severe degree of pericardial effusion and hence to be at risk of hemodynamic instability in the absence of interventions. Therefore, close monitoring and early prescription of anti-inflammatory treatments are therefore warranted in symptomatic patients. Data on the efficacy of anti-inflammatory drugs for postpericardiotomy syndrome in children is limited. While aspirin has been widely used, the reports were largely unreliable (4) . to date, there have been no controlled trials on the efficacy of non-steroidal anti-inflammatory agents. Hugh et al. (1) recommended the 100 mg/kg/day dose of aspirin for post-op PEs in four divided doses for about one month, then tapering over one to two months. We found aspirin effective (77 % response) even at lower doses and shorter duration. The recommended dose of prednisone by Hugh et al. (1) is 2 mg/kg/day for one week and gradually tapered over two to three weeks. We got a 90 % response with a half dose of their recommendation in nearly the same duration. In a double blind placebo controlled trial of steroids in a small cohort of children with postpericardiotomy syndrome, Wilson et al. (9) showed that steroids accelerates the remission in the majority, whereas two of their 12 patients had a further increase in the amount of effusion despite steroids administration. Likewise, only three of our 41 (87.3 %) patients failed to respond to an anti-inflammatory treatment. Therefore a trial anti-inflammatory treatment seems to be essential in patients with moderate and increasing amounts of pericardial effusion before contemplation of more invasive pericardiocentesis. There are few studies examining the risk factors predisposing to development of pericardial effusion after heart surgeries for congenital heart diseases. Cheung et al. (10) suggested a higher incidence of pericardial effusion after Fontan-type procedures compared with other cardiac surgeries. Likewise, we found that pericardial effusion occurred more commonly after Fontan-type procedures and AVSD repair (29 %, 5 of 17 for both). Multivariate analysis showed that postoperative warfarin may be an additional risk factor after adjustment for other variables. As we routinely use warfarin in post op patients after Glenn shunt, Fontan-type procedures and valve replacements, our data is insufficient to conclude that warfarin is an independent risk factor. Cheung et al. (10), found female sex as an independent determinants and a risk factor for PE, but in our study we didn`t find any significant difference between genders.

Based on our findings, serial echocardiography up to one month after selected at-risk patients may be needed, namely those with symptoms suggestive of postpericardiotomy syndrome and those after Fontan-type procedures and AVSD repair. Incidence of PE after palliative heart surgeries is considerable; following such patients for PE should not be forgotten. Low doses of aspirin and corticosteroids may be effective in management of post-op pericardial effusions. A well designed Multi-center study could be helpful to bypass some study limitations and to provide a guideline for the better approach to Post Pericardiotomy Effusion in children. We treated almost all patients with pericardial effusion and perhaps, some small effusions would have resolved without any therapy itself. ASA or corticosteroids prescription was based on physician preference, which raises the question that the treatment strategy should have been randomized and blinded or not. Further discussion against data efficacy may be useful. Also, we did not study the standard doses recommended, thus this still leaves the question whether treatment efficacy could be better especially in the aspirin group.

Residual shunts and valvular dysfunctions may interfere with the occurrence of post-operative PE. We didn’t compare the prevalence of PE in patients with and without residual shunts and valvular dysfunctions.
